# Feasibility of Using High-Resolution Computed Tomography Features for Invasiveness Differentiation of Malignant Nodules Manifesting as Ground-Glass Nodules

**DOI:** 10.1155/2022/2671772

**Published:** 2022-10-17

**Authors:** Xinyue Chen, Benbo Yao, Juan Li, Chunxiao Liang, Rui Qi, Jianqun Yu

**Affiliations:** ^1^CT Collaboration NE Asia, Siemens Healthineers, Chengdu, China; ^2^Department of Radiology, Zigong Fourth People's Hospital, Zigong, China; ^3^Department of Radiology, West China Hospital, Sichuan University, Chengdu, China

## Abstract

Ground-glass nodule (GGN)-like adenocarcinoma is a special subtype of lung cancer. The invasiveness of the nodule correlates well with the patient's prognosis. This study aimed to establish a radiomic model for invasiveness differentiation of malignant nodules manifesting as ground glass on high-resolution computed tomography (HRCT). Between January 2014 and July 2019, 276 pulmonary nodules manifesting as GGNs on preoperative HRCTs, whose histological results were available, were collected. The nodules were randomly classified into training (*n* = 221) and independent testing (*n* = 55) cohorts. Three logistic models using features derived from HRCT were fit in the training cohort and validated in both aforementioned cohorts for invasive adenocarcinoma and preinvasive-minimally invasive adenocarcinoma (MIA) differentiation. The model with the best performance was presented as a nomogram and was validated using a calibration curve before performing a decision curve analysis. The benefit of using the proposed model was also shown by groups of management strategies recommended by The Fleischner Society. The combined model showed the best differentiation performance (area under the curve (AUC), training set = 0.89, and testing set = 0.92). The quantitative texture model showed better performance (AUC, training set = 0.87, and testing set = 0.91) than the semantic model (AUC, training set = 0.83, and testing set = 0.79). Of the 94 type 2 nodules that were IACs, 66 were identified by this model. Models using features derived from imaging are effective for differentiating between preinvasive-MIA and IACs among lung adenocarcinomas appearing as GGNs on CT images.

## 1. Introduction

Screening by imaging is important for the early detection of lung cancer at a curable and manageable stage to reduce the mortality rate [1]. As for an effective screening, on which cohort it is performed is important. In the United States, the heavy smoking cohort was selected by the National Lung Screening Trial (NLST), as smoking is a major risk factor for lung cancer [2]. Unlike the United States, an increasing incidence of lung cancer has been noted among women who are nonsmokers, particularly in Asia [3]. This might be due to the variation in genetic factors across regions [3]. A modified criterion to conduct screening in such regions might include patients outside the NLST. Besides, along with the advent of low-dose CT with fewer adverse effects, a relatively larger number of nodules with different degrees of invasiveness is expected to be pathologically found but they have a similar appearance as CT. Radiomic is of great clinical significance in the decision-making process involved in such scenarios [4].

Ground-glass nodule (GGN)-like lung adenocarcinoma is a special subtype of lung cancer [5]. It is different from conventional lung adenocarcinoma, as its ground-glass component is an indolent indicator [6]. A study showed that in GGN-like lung adenocarcinoma, the features correlated with malignancy and prognosis change from one category to another as defined in radiologic imaging in an average of ≥2 years [7]. Furthermore, only 4% of those initially defined as pure GGNs were pathologically deemed as invasive adenocarcinomas (IACs) after surgery [8]. Compared with early lung cancer with surgical resection as the standard of care [9], conservative observation seems to be appropriate for GGN-like lung adenocarcinoma. Moreover, the current choice of treatment and extent of surgical resection, including lobectomy or sublobular resection, for this particular type of lung cancer remains controversial owing to insufficient data [5]. Therefore, physicians should be more cautious in selecting surgical procedures. Conversely, although The Fleischner Society is involved in continuously updating the follow-up strategy and the accumulated evidence and experience [10], patient preference plays a significant role in clinical decision-making. In 2017, Sawada et al. reported that 73% of the lesions in their study were resected without any evidence of progression [8].

In 2011, the International Association for the Study of Lung Cancer, American Thoracic Society and European Respiratory Society (IASL/CATS/ERS) proposed a new histological classification for lung adenocarcinomas, which had four categories: preinvasive lesions, minimally invasive adenocarcinoma (MIA), IAC, and variants [11]. These categories were further divided into three major groups of grades of behavior, with preinvasive and minimally invasive lesions being categorized in the same group [12]. These classifications are well correlated with the prognosis. As high-resolution computed tomography (HRCT) becomes the standard diagnostic procedure for lung cancer [13], it is especially important in the classification of GGN-like adenocarcinoma because the subtypes divided by radiological features were demonstrated to be relevant to prognosis [7]. In this study, from the radiologists' perspective, we attempted to establish a closer connection between the images and histological invasiveness of GGN-like lung adenocarcinoma. If built successfully using relevant quantitative imaging features, such models could allow accurate invasiveness estimation of GGNs at the individual level. It would be of considerable significance in guiding the decision-making of physicians, for instance, in determining the timing of the initial follow-up, appropriate follow-up duration, and the best surgical strategy in a noninvasive manner.

## 2. Materials and Methods

This retrospective study was approved by the Biomedical Research Ethics Committee of our institution, and the requirement for written informed consent was waived. Moreover, this study was conducted in accordance with the transparent reporting of a multivariate prediction model for individual prognosis or diagnosis (TRIPOD) statement [14].

### 2.1. Data Source and Eligibility Criteria

In our radiology information system, we retrospectively searched for patients from January 2014 to July 2019, who had pulmonary nodules manifesting as ground-glass opacities (GGOs) on preoperative HRCT and were diagnosed with clinical stage 1A lung adenocarcinoma or its precursor. Subsequent surgery was performed as the treatment option within a month after HRCT. The postoperative pathological results of malignant nodules based on the classification introduced by IASL/CATS/ERS in 2011 were obtained. The exclusion criteria were as follows:

1. GGNs with a diameter >3 cm
2. Preoperative CT with a slice thickness >1.5 mm and/or without a high-resolution mode
3. GGNs with benign postoperative pathological results

All thoracic CT acquisitions were performed on a second-generation, dual-source, dual-energy CT scanner (SOMATOM Definition Flash, Siemens Healthineers, German). Noncontrast CT images were included in the study. The standard thoracic scan protocol was used with the following scanning parameters: tube voltage, 120 kVp; pitch, 0.85; and slice thickness, 1 mm. An automated, real-time, attenuation-based tube current modulation software (CARE DOSE 4D) was used to control radiation exposure. The images were then reconstructed with a thickness of 1.5 mm using D36f as a kernel for mediastinal images and D70f for lung images.

### 2.2. Groups, Outcomes, and Predictors Analyzed

Three types of nodules were collected with respect to three different follow-up strategies recommended by The Fleischner Society [10]. Specifically, nodules <6 mm (hereafter referred to as type 1) were all pure GGNs. Nodules, either pure or part-solid, were found to have a diameter of ≥6 mm and the solid component had a diameter of <6 mm (type 2). Nodules were all part-solid GGNs, with the solid component having a diameter of >6 mm (type 3). The pathologic diagnoses for these nodules are as follows: (1) atypical adenomatous hyperplasia (AAH); (2) adenocarcinoma in situ (AIS); (3) MIA; and (4) IAC. Moreover, the proposed model attempted to differentiate IAC from the other pathologic types of adenocarcinoma.

Two radiologists, both with >5 years of experience in chest CT reading, performed the following semantic feature evaluation. GGOs were identified as areas of increased attenuation without obscuring the normal lung structures. The nodules composed of 100% GGOs based on HRCT were defined as pure GGNs, whereas the others were classified as part-solid GGNs.

The other qualitative semantic features of nodules, besides the types (pure GGNs or part-solid GGNs), included margins (smooth, lobular, or spiculated), internal features (presence of air bronchial signs), and adjacent structures (presence of the pleural tag and vascular convergence). The diameter was measured as the average of long and short axes on the transverse images of the lung window. In case of disagreement between the radiologists, a consensus was achieved by joint assessment.

For texture quantitative feature calculation, the nodule would be segmented first. All CT images were loaded in a dedicated prototype (Radiomics, Siemens Healthineers) for processing. The radiologists identified and segmented the respective nodules using the same method used for lesion adaptation provided by the prototype was used for rough nodule segmentation [15]. A drawing tool (nudging and brushing) was then used for slice-by-slice segmentation refinement to ensure that correct lesion volume boundaries are obtained on both the lung and mediastinal windows of images [15]. Then, the three-dimensional (3D) volumes of interest (VOIs) were created for subsequent feature computations.

Subsequently, the same prototype (Radiomics) interfacing with PyRadiomics was used for feature computation from the VOIs [15]. Before computation, the images were preprocessed using different Laplacian effects for Gaussian or wavelet filtering. Then, the feature classes, including first-order, shape, and texture classes (gray level dependence matrix; gray level co-occurrence matrix; gray level size zone matrix; and neighboring gray tone difference matrix), were calculated for both original and processed VOIs. A total of 1319 texture features were computed from each VOI.

### 2.3. Predictive Modeling

**Figure 1:**
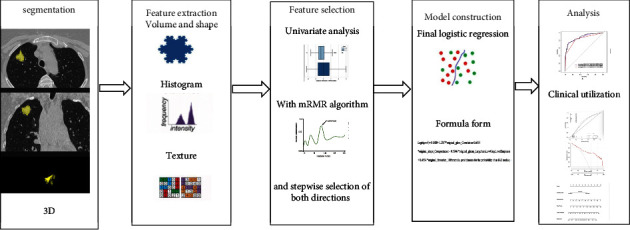
Flowchart of texture modeling process.

In this study, semantic, texture, and combined models were built. The training cohort was used to fit the model. As for feature selection of the texture model, univariate statistics were used. First, all 1319 texture features were ranked based on their explanatory powers for differentiation, which was defined by an R2 value. After conducting a false discovery rate Benjamini–Hochberg correction for multiple comparisons, a *p* value <0.05 was considered statistically significant. Then, a fast-minimum redundancy maximum relevance (mRMR) algorithm was used to select a set with a predefined maximum number of features. In this study, the maximum number was set to 10. Multivariable logistical regression modeling was applied to the selected features using forward selections based on the Akaike information criterion (AIC). The flowchart for the texture modeling process is shown in Figure1.

For semantic feature selection, all invasiveness-relevant features were included in the multivariate logistical regression. A combined model was also built using both semantic and texture information.

All three models were validated in the testing cohort and training cohort with 50 resamples from 5 repeats of 10-fold cross-validation. A nomogram for model visualization was drawn for the model with the highest differentiation ability. The clinical benefit was illustrated for this model by decision curve only in the testing cohort after calibration curves. The apparent benefit was demonstrated on a jitter plot for the three types of nodules grouped by the follow-up strategies recommended by The Fleischner Society based on the diameter.

### 2.4. Other Statistical Analyses

Numerical variables were summarized as mean ± standard deviation or median (interquartile range). Categorical variables were expressed as numbers (%). In the comparison between groups, an unpaired *t*-test or the Mann–Whitney *U* test was used for numerical variables. For categorical variables, a chi-square test or Fisher's test was used when appropriate. The Wald test was used for regression parameter analysis. Receiver operating characteristic (ROC) curve analysis was applied for model performance evaluation in terms of the area under the curve (AUC), with bootstraps to determine the confidence intervals (CIs). A DeLong test was used to compare the AUCs of the two models. The confusion matrix was also calculated using 0.5 as the cut-off value. For the model goodness of fit testing, the Hosmer–Lemeshow test was used. A *p*value of <0.05 (two-tailed) was considered statistically significant. Prism 8 and R language software programs were used in the analyses.

## 3. Results

### 3.1. Characteristics of the Cohort Analyzed

**Figure 2:**
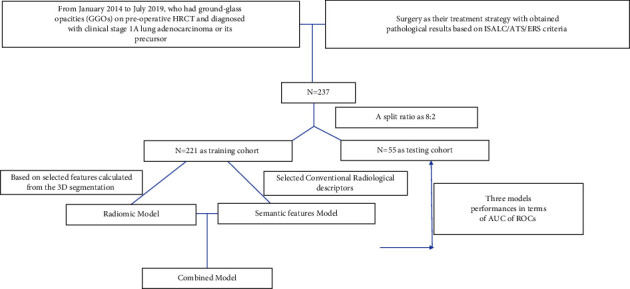
Nodules on HRCT enrolment of modeling.

Eventually, 237 patients (74 males and 163 females) were enrolled in the study (age, 55.0 ± 11.2 years), and a total of 276 GGNs were collected. There were 15 type 1, 208 type 2, and 55 type 3 nodules. The pathological diagnoses for these nodules were AAH (*n* = 24), AIS (*n* = 23), MIA (*n* = 83), and IAC (*n* = 145). The proposed model tried to differentiate IAC from other pathologic types of adenocarcinomas. A stratified random splitting method was applied to split the total number of nodules in an 8:2 ratio into training (*n* = 221) and testing (*n* = 55) cohorts (Table 1). The enrolment of the models is presented in Figure 2.

### 3.2. Model Building and Performance

#### 3.2.1. Demographic and Semantic Feature Selection

Between our training and testing cohorts, both age (age, 54.9 ± 10.80 years and 55.6 ± 10.89 years, respectively; *p*=0.69) and gender (female, 70.1% and 78.2%, respectively; *p*=0.24) were not significantly different.

Three features (nodule type, lesion margin, and pleural indentation) from six collected semantic features demonstrated a significant relevance with histological invasiveness types (pre-MIA vs invasive) in both training and testing cohorts (all *p* < 0.05). The association with vascular convergence was significant in the training cohort (*p* < 0.0001) but not in the testing cohort (*p*=0.11). Regarding the feature of air bronchial signs, no significant correlations were found (both *p* > 0.05).

**Figure 3:**
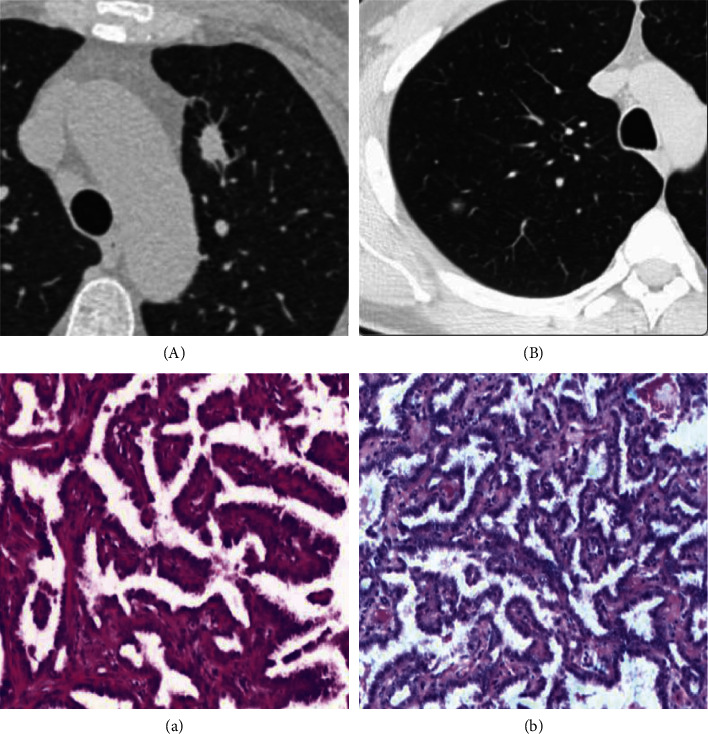
Correlation of nodule margin with the pathological results. Nodules were shown on HRCT with spiculation margin (A) and smooth margin (B), and their corresponding pathological results were ICA (a) and AAH (b).

Univariate logistic regression models were built for three significant features. After applying the ROC analysis, the lesion margin showed the highest differentiation ability for both training and testing cohorts (AUC = 0.78 and 0.75, respectively). Pleural indentation showed an AUC of 0.71 for the training cohort and 0.67 for the testing cohort. The nodule type had a comparable AUC in the training and testing cohorts, with an AUC of 0.67 (Table 1). The correlation between semantic features and the pathological results is presented in Figure 3.

#### 3.2.2. Semantic Model Building

After adding these three features into one multivariate logistic model, only pleural indentation and lesion margin were found to be independently significant. The two significant features were used for the subsequent semantic regression model building, that is, Logit (prob) = −1.414 + 2.700^*∗*^pleural indentation + 1.953^*∗*^ lesion margin. Here, prob denotes the probability of an IAC nodule.

#### 3.2.3. Texture Feature Selection and Modeling

**Figure 4:**
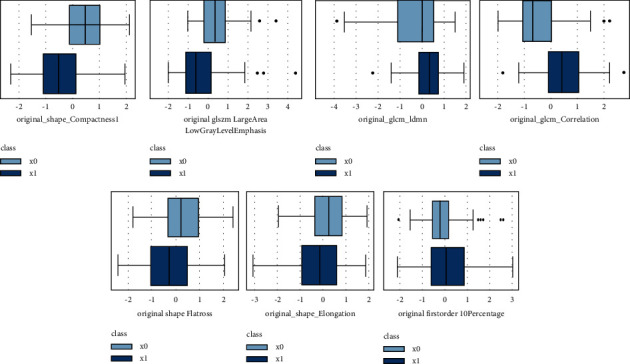
Boxplots of value distribution for five selected quantitative texture features derived from HRCT.

After using RMR algorithms for univariate analyses, a set of features, including original_glcm_correlation, original_shape_compactness, original_glszm_largearealowgraylevelemphasis, original_glcm_idmn, original_shape_flatness, original_shape_elongation, and original_firstoder_10 percentage were selected for subsequent multivariable logistic regression modeling (Figure 4).

After conducting forward selection based on AIC and using multivariable logistic regression, the final model was built and selected as follows:

Logit (prob) = 0.1580 + 1.2387^*∗*^original_glcm_correlation-0.6595^*∗*^original_shape_compactness1—0.5846^*∗*^original_glszm_largearealowgraylevelemphasis +0.4534^*∗*^original_firstorder_10percentile. Here, prob denotes the probability of an IAC nodule.

#### 3.2.4. Combined Model Building

The combined model was built based on the logistic regression with two variables. They were the estimated probabilities of IAC calculated by the semantic and texture models. The formula for this model is provided in the following equation: Logit (prob) = −3.139 + 3.623 × prob estimated by the texture model + 2.713 × prob estimated by the semantic model, where, prob denotes the probability of an IAC nodule. The inferential results of this model showed that both variables were independently correlated with the label (both *p* < 0.001).

#### 3.2.5. Model Performance Validation

**Figure 5:**
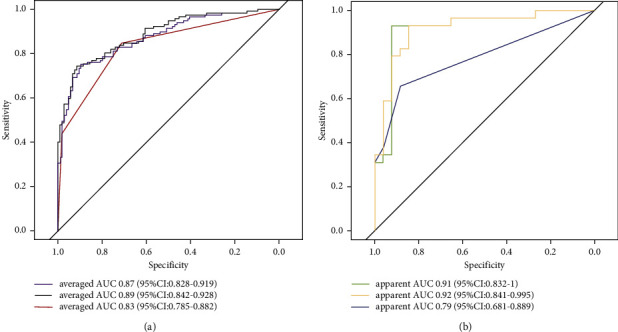
ROC curves of invasiveness predictive models. (a) Averaged ROC tested in 50 resamples of the training cohort using 5 repeats of 10-fold cross-validation. (b) ROC tested in our testing cohort. The purple and green lines are for the texture model, the black and orange lines are for the combined model, and the red and blue lines are for the semantic model.

The semantic model had an AUC of 0.83, which was lower than that of the texture model, with an AUC of 0.87 and the combined model with an AUC of 0.89. These were validated using 50 resamples from the training set. A similar pattern was shown by the results in the testing cohort, with the highest AUC of 0.92 for the combined model, 0.91 for the texture model, and 0.79 for the semantic model. The difference between the AUCs of the semantic and the combined model was statistically significant, as shown in the testing and training cohorts (both *p* < 0.05). All models showed comparable performance validated by the testing and training cohorts (*p*=0.38 for the texture model, 0.42 for the semantic model, and 0.48 for the combined model) using a DeLong test for comparing the differences in AUCs (Figure 5 and Table 2).

#### 3.2.6. Benefit of Using the Best Model in Clinical Practice

**Figure 6:**
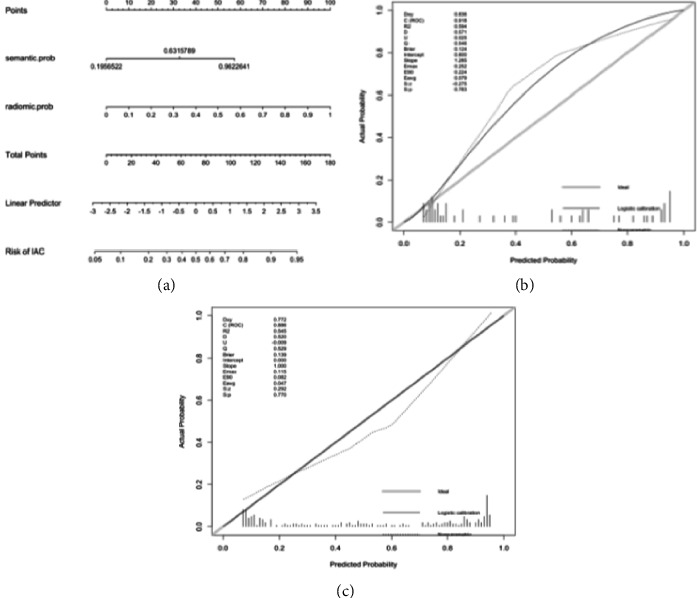
Nomogram of the combined model constructed in our training cohort. The semantic and texture signatures were incorporated as factors (a). The calibration curves show a good agreement between the model predicted and actual probability of being an invasive nodule in the training cohort (b) and testing cohort (c).

**Figure 7:**
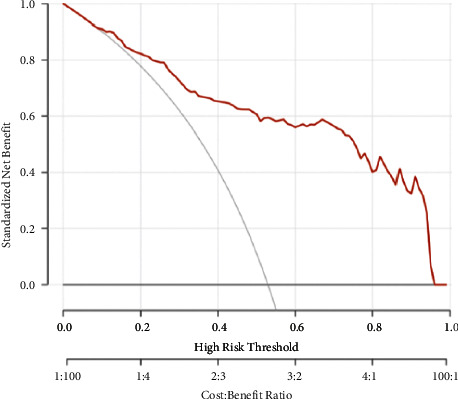
Decision curve tested using all the nodules. It shows higher net benefit across all the risk threshold compared to treat-all and treat-none strategies.

A nomogram was constructed (Figure 6). Both curves (logistic regression and loess smoother) were close to the ideal 45° line for both datasets, which showed a visual impression of good calibration (Figure 6). The Hosmer−Lemeshow test showed that the model had a good fit (*p*=0.16). As shown in the decision curve (Figure 7), when the doctors' experience was used in a trade-off between the benefits and harms of the treatment, additional guidance by the model achieved higher net benefits than treat-all or treat-none strategies for all patient groups, with either conservative or aggressive treatment decisions.

#### 3.2.7. Apparent Benefit Demonstration

**Figure 8:**
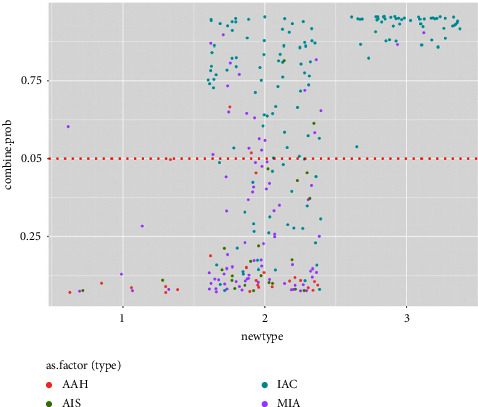
Jitter plot of nodule classifications with their model predictive values. It illustrates that using a model predictive value of 0.5 (shown with a red dot) as the cut-off, a correct reclassification with respect to histological invasiveness would be identified, especially for type 2 nodules.

None of the type 1 nodules were IACs; 14 of them were correctly classified by the proposed model. Out of 208 type 2 nodules, 94 were histologically proven to be invasive adenocarcinomas and 66 were detected by the proposed model. For the type 3 nodules, 51 of 53 were IACs, and they were all successfully identified by the proposed model (Figure 8).

## 4. Discussion

GGNs on HRCT were found to be the common manifestations of lung cancer, but pathologic findings may be preinvasive or invasive. It is helpful for clinical decision-making if there were some models to predict the invasiveness of lung cancer based on its features on HRCT images. The current study showed that either semantic or texture features on HRCT images correlated with the histological invasiveness of GGN-like adenocarcinomas, and combining these two features in a model leads to the highest performance (AUC = 0.92).

The nodule type, lobulated or speculated edge, and pleural tag were stable invasiveness-relevant semantic features. The margin type showed the highest differentiation ability, with an AUC of 0.78 for the training cohort and 0.75 for the testing cohort. In a study on imaging findings for GGN malignancy prediction in 2009, the margin was also shown to be relevant [16]. From a histological perspective, the fibroblastic stromal invasion might be the cause of the lobulated tumor margins [17]. The other two signs showed a moderate differentiation performance, which was consistent with the results of the meta-analysis conducted by Dai et al., with a comparable ability when the feature was used alone [18]. The margin and pleural tag were independent predictors based on multivariate regression modeling. Similar findings were also reported by Xue et al. [19]. By combining these two signs in the current study, the differentiation ability was increased to 0.83 for the training cohort and 0.79 for the testing cohort.

The role of diameter for the purpose of differentiation has been elucidated by many studies. In 2013, Lee et al. found that a 10-mm cutoff diameter for preinvasive type identification in pure GGNs has 100% specificity, which is extremely useful for physicians seeking positive evidence [20]. They used the maximal diameter in the transverse view for size estimation. However, as suggested by The Fleischner Society in 2005, the average of long and short axes would allow determining a more accurate 3D tumor volume [21]. We used this averaging method for diameter measurement. To provide a model with an effective differentiation ability regardless of the diameter, the diameter was not enrolled as a variable in the proposed model. Notably, we showed the benefit of the proposed model in groups based on diameter for different management strategies recommended by The Fleischner Society in 2017 [10].

For the texture model, we used a dedicated semiautomatic 3D segmentation prototype embedded with a Python-based texture feature calculator (Radiomic, Siemens Healthineers, Germany). The linear regression model was fitted with five selected texture features with high discrimination ability, with an AUC of 0.87 for the training cohort and 0.91 for the testing cohort. The discrimination ability was higher in the texture model than in the semantic model (AUC, training cohort = 0.83, testing cohort = 0.79). This might be due to the continuous nature of texture features, whereas semantic features have a binary nature. The effectiveness of continuous texture features in invasiveness modeling was also shown in studies. Sun et al. built a model with an AUC of 0.72 for invasiveness identification in 2020 [22]. They dichotomously classified MIA and invasive adenocarcinoma in one group and the others in another group. However, as shown in a study on the correlation of histological classification with prognostic subgroups, MIA and preinvasive nodules were found to be in the same grade group with similar behavior, which was different from that observed for invasive nodules [12]. The current classification method might be more practical. We excluded the benign nodules despite the fact that these types added more variation. We found two studies with the exact same grouping criteria, and all of them used the linear modeling method [23, 24]. Compared with these studies, the proposed model was trained with the highest number of data (*n* = 221). Besides this, the image inputs for the model were unique with a high-resolution feature, that was reconstructed by the kernel with less smoothing degree and thus it presented more details. These might account for the result that the proposed model showed the highest performance of 0.89 versus 0.86 (Zheng et al) and 0.862 (Wu et al). A recently published paper in 2022 [25] attempted to use the more complex nonlinear model to build the correlation between images and the differentiation aim. They also used high-resolution images as inputs. Notably, they achieved an AUC of 0.978 using their data. However, their data size was small (*n* = 168). The uncertainty and overfitting of the result might be suspicious, especially when considering the highly flexible nature of the nonlinear modeling algorithm. We wish to validate the model using our data. However, this nonlinear model lacks details. Besides, it could not be presented as a nomogram or as a formula form, like ours, for an easy implement in clinical practice and for our intended validation.

We added the estimated probability from both semantic and texture models into a single regression model. The result showed that these two factors independently correlated with the label, implying that both variables contributed to the model in different ways. The AUC was further increased to 0.89 and 0.92 for the training and testing cohorts, respectively. A probability calibration test was performed. The model showed the goodness of fit. The decision curve showed a higher clinical gain of net benefit based on the model-suggested probability of invasiveness. Finally, we compared the benefit of using the proposed model on the three types of nodules. According to The Fleischner Society, routine follow-ups were not recommended for patients with type 1 nodules, and more aggressive follow-up strategies, including surgical intervention, were recommended for patients with type 3 nodules. The results showed high accuracy of the model in each type. We could infer that these type 2 nodules, which had the largest sample size and highest variation in invasiveness degree, would benefit the most by using the proposed model.

The software in this study allows for a more efficient workflow on a cohort basis. It contains three functional segments for segmentation, feature selection, and integrated statistical analysis. The workflow is as follows: (1) with 3D medical images in a cohort as input entity, multiple segmentation tools could be applied for user-defined VOIs creation; (2) features based on the standard PyRadiomic library were computed for VOIs; and (3) calculated features for each sample in the cohort were tabulated for subsequent statistical and machine learning analysis. The ready-to-use analysis methods include clustering, univariable statistics, multivariable statistics, and the machine learning algorithm. It is a one-stop solution that integrates algorithms for both image and numerical data analysis in one user-friendly interface. And it provides three types of outputs: segmentations, tabulated features, and statistical result tables and plots. It makes radiomic research studies possible even for the less experienced researcher.

This study has two limitations. First, we did not make a conclusion for the model usage, whether for aiding surgical interventions or follow-up strategies. This was mainly because we only investigated the correlation between the images and the histological invasiveness of the nodules. An aspect regarding the growth potential should be considered in the final clinical decision for a nodule. Thus, we will study the probability of growth prediction by imaging in the future. The model was specifically fit for malignant nodules, but its performance for GGNs, including both benign and malignant nodules, remains unknown.

## 5. Conclusion

In this study, we successfully built a model with a high performance (AUC = 0.92) using semantic and texture features for the histological invasiveness prediction of malignant GGNs preoperatively and noninvasively. The revised guideline for GGNs provided greater discretion to accommodate individual risk factors and preferences. As the model is convenient to use for combining multiple factors and validating an independent dataset to be a reliable way for risk estimation at the individual level, it is of great clinical significance.

## Figures and Tables

**Table 1:** Patient demographic information comparison between the training and testing cohorts and semantic imaging feature comparison between the invasiveness subtypes and differentiation ability in terms of AUC.

Patient demographical information								
	Training			Testing			*p* value	

Age	54.9 (±10.80)			55.6 (±10.89)			0.69	
*Gender*							0.24	
Female	155 (70.1%)			43 (78.2%)				
Male	66 (29.9%)			12 (21.8%)				

*GGO characteristic*								
	Training		*p* value	AUC	Testing		*p* value	AUC

	Pre-MIA	Invasive-			Pre-MIA	Invasive-		
Nodule type			<0.0001^*∗*^	0.67			0.0009^*∗*^	0.67
Pure	102 (98.08%)	75 (64.1%)			26 (100%)	19 (65.52%)		
Part-solid	2 (1.92%)	42 (35.90%)			0 (0%)	10 (34.48%)		
Lesion margin			0.02^*∗*^	0.78			<0.0001^*∗*^	0.75
Smooth	74	18			24	12		
Lobular or spiculated	30	99			2	17		
Air bronchial sign present	3 (2.88%)	8 (6.84%)	0.18		2 (7.69%)	4 (13.79%)	0.67	
Pleural indentationpresent	2 (1.92%)	51(43.52%)	<0.0001^*∗*^	0.71	1(3.85%)	11 (37.93%)	0.0027^*∗*^	0.67
Vascular convergence present	1 (0.96%)	32 (27.35%)	<0.0001^*∗*^		0(0%)	4 (13.79%)	0.11	
DGGN	10.01 (±4.65)	15.38 (±7.22)	<0.01^*∗*^		18.11 (±8.80)	9.32 (±2.45)	<0.0001^*∗*^	
GGN<10 mm	62	11			15	3		
GGN>10 mm	42	106			11	26		

^*∗*^ *p* < 0.05.

**Table 2:** Detailed diagnostic performance of the three models in the training and testing cohorts.

	*Semantic model*		*Texture model*		*Combined model*	
	Training cohort	Testing cohort	Training cohort	Testing cohort	Training cohort	Testing cohort
Sensitivity	84.6% (95%CI: 78.1%–91.2%)	65.5% (95%CI: 48.2%–82.3%)	78.6% (95%CI: 71.2%–86.0%)	82.8% (95%CI: 69.0%–96.5%)	79.5% (95%CI: 72.2–86.8%)	79.3% (95%CI: 64.6%–94.1%)

Specificity	71.2% (95%CI:62.4%–79.9%)	88.4% (95%CI: 76.2%-1)	75.0% (95%CI: 66.7%–83.3%)	92.3% (95%CI: 82.1%-1)	78.8% (95%CI: 71.0%–86.7%)	92.3% (95%CI: 82.1%-1)

PPV (%)	76.7% (95%CI:69.5%–84.0%)	86.4% (95%CI: 72.0%-1)	78.0% (95%CI: 70.5%–85.4%)	92.3% (95%CI: 82.1%-1)	80.9% (95%CI: 73.7%–88.1%)	92.0% (95%CI: 81.3%-1)

NPV (%)	80.4% (95%CI:72.3%–88.5%)	69.7% (95%CI: 54.0%–85.4%)	75.7% (95%CI: 67.4%–84.0%)	82.8% (95%CI: 69.0%–96.5%)	77.4% (95%CI: 69.4%–85.3%)	80.0% (65.7%–94.3%)

## Data Availability

The image data together with computed features used to support the findings of this study are available from the corresponding author upon request.

## Abbreviations

GGNS: Ground-glass opacity nodules
IAC: Invasive lung adenocarcinoma
MIA: Minimal invasive adenocarcinoma
AUC: Area under curve
ROC: Receiver operator curve

## Additional Points

The link between CT and histological invasiveness for GGNs has been found. We successfully quantified the correlation using linear models. Quantitative features were better in differentiation than semantic descriptors. Management for patients might become more appropriate with the model.
